# Reducing Emergency Medical Services (EMS) Usage as Interfacility Transport for Patients Presenting with Chest Pain

**DOI:** 10.3390/jcm15041462

**Published:** 2026-02-13

**Authors:** Mark Keith Hewitt, Alisha Greer, Shawn Mondoux

**Affiliations:** 1 Department of Emergency Medicine, Health Sciences North, Sudbury, ON P3E 5J1, Canada; 2 Department of Critical Care Medicine, Health Sciences North, Sudbury, ON P3E 5J1, Canada; 3 Division of Clinical Sciences, NOSM University, Sudbury, ON P3E 2C6, Canada; 4 Department of Medicine, Division of Emergency Medicine, McMaster University, Hamilton, ON L8S 4L8, Canada; 5 St. Joseph’s Healthcare Hamilton, Hamilton, ON L8N 4A6, Canada

**Keywords:** chest pain, interfacility transport, data drilldown, quality improvement, emergency medical services, cardiac monitoring

## Abstract

**Background**: Acute coronary syndrome (ACS) is a “can’t miss” diagnosis. The gold-standard workup for this requires serial troponin biomarker evaluation over a period of hours. Traditionally, many of these patients required telemetry while being evaluated in this fashion; however, the high-quality literature suggests that low-risk patients do not require ongoing continuous cardiac monitoring. Locally, it was found that over 70% of patients presenting with low-risk chest pain to our high-volume urgent care were transferred to the main hospital for an ACS rule-out work-up via emergency medical services (EMS). We felt this intersection of patient care and medical services could be streamlined to reduce critical resource utilization. **Objective**: The aim of this study is to reduce the usage of EMS utilization for transport of low-risk chest-pain patients from the urgent care to the main hospital by 25% over a 3-month period. **Methods**: This study was conducted as an uncontrolled before–after interrupted time series design. A comprehensive data drilldown was performed through a chart review and structured clinical-practice evaluation. This led to a multi-factorial quality improvement initiative centered around the creation of an evidence-based safe-for-self-transport tool and physician education. The primary outcome measure was the proportion of patients transported via EMS with the main balancing measures being the proportion of self-transported patients admitted to the hospital and the time to troponin blood-draw in self-transported patients. **Results**: The education and the newly developed transport tool resulted in a sustained shift below the previous baseline system mean control limit, indicating a significant reduction in EMS usage for patient transport. The overall reduction in usage was 30%. No change in balancing (safety) measures was identified post-implementation. **Conclusions**: EMS remains a finite resource within many Canadian health regions. The results of this study show that by focusing on a cardinal emergency-department presentation like chest pain, adapting evidence-based practice through quality-improvement methodologies can result in a significant sustained reduction in EMS utilization.

## 1. Introduction

Acute coronary syndrome (ACS) is a “can’t miss” diagnosis. While there are no specific criteria for who requires a diagnostic work-up for this pathology, most adult patients presenting with chest pain to an emergency department (ED) will require this diagnosis to be “ruled-out” before discharge. The use of serial troponin biomarkers to aide in this diagnosis has become the standard of care when assessing patients [1]. The American Heart Association suggests that two serial measurements of a troponin three hours apart be used before determining if a patient is low-risk and ready for discharge [1].

Patient factors, previous disease, and serial electrocardiogram (ECG) can aide in this disposition planning for further inpatient versus outpatient investigation of these patients. Risk stratification scoring systems such as the Heart score, clinical chemistry score and other clinical decision tools (CDT) aide in the ability to discharge these patients from the emergency department [2,3,4]. Further streamlining this care process is a growing body of high-quality literature suggesting that patients possessing an ECG with no signs of ischemia and either normal initial troponin value or resolved chest pain do not require ongoing continuous cardiac monitoring (CCM) throughout the remainder of their evaluation [1,5,6]. Furthermore, the utility of CCM for arrythmia in low-risk chest pain remained in question before these modern studies, citing low event rates in the lower-risk chest-pain population [7,8,9,10]. This becomes a clinically important consideration when evaluating patients for placement in overcrowded emergency departments or facilitating transport from urgent care/outpatient facilities.

Local data suggests that only 1.1% of these chest-pain patients presenting to our local urgent care center (UCC) with initially negative cardiac markers go on to have a positive change at three hours necessitating admission. Despite this, over 70% of them are transported by an ambulance to the affiliated hospital, citing a need for CCM as the main reason. While there is no direct comparison of Emergency Medical Services (EMS) over-usage for interfacility transport in Canada, the National Hospital Ambulatory Medical Care Survey in the United States indicates that at least 13–17% of all EMS transport is medically unnecessary in all-comers [11]. Furthermore, local data suggests that only 20% of these patients are offloaded to a hospital stretcher within 30 min of arrival to the main hospital, further tying up EMS resources. With an additional transport time on average of 30 min per patient (local data) this represents a significant burden on the local EMS availability, potentially limiting community access to care. Therefore, the aim of this study is to reduce the usage of EMS for the transport of chest-pain patients from the urgent care to the main hospital by 25% over a 3-month period.

## 2. Methods

### 2.1. Context

This improvement project was carried out at a large tertiary care, inner city academic hospital system within Ontario, Canada with an average census of 65,000 visits per year. The adjacent UCC for this hospital sees an additional 65,000 patients per year. The same physician group regularly rotates through both sites as part of their clinical commitments.

### 2.2. Study Design

This study was conducted as an uncontrolled before–after interrupted time series design. The retrospective chart access for audit and data collection was approved by the Hamilton Integrated Research Ethics Board (HIREB 12865-C) and quality improvement (QI) exemption was granted for ongoing prospective data collection through the duration of this study.

The first phase of the study focused on baseline data acquisition of the specific chest-pain population presenting to the UCC for an 8-month inclusive sample period. Inclusion criteria are as follows:

- Age over 18 presenting to UCC;
- Serial troponin testing for the purposes of ACS rule-out;
- ECG acquisition.

### 2.3. Main Intervention

**Figure A1:**
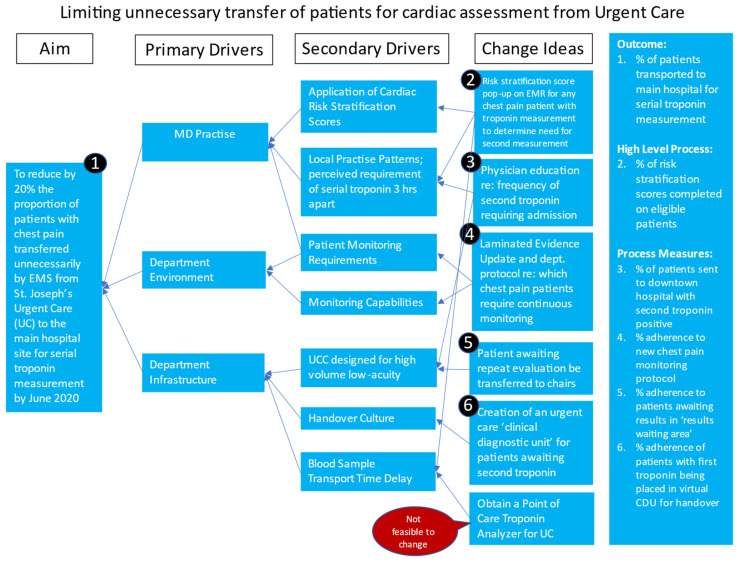
Driver diagram for chest-pain transport. Summation of root-cause and thematic analysis with probable etiology’s for transport by EMS. 1—represents overall goal and idea with numbers 2–6 representing potentially feasible change ideas.

The second phase of this study consisted of stakeholder engagement in root-cause analysis interviews to further delineate perspective on practice patterns and departmental flow. A driver diagram (Appendix A, Figure A1) is an illustrative representation of all root-causes identified for potential interventions.

Focused development and implementation of a “safe-for-self-transport” Clinical Decision Tool (CDT) was pursued following identification of the perceived need for continuous cardiac monitoring in this population as a consistent driver for EMS transport of this patient population.

#### 2.3.1. Developmental PDSA—Chest-Pain Monitoring Transport Decision Tool

**Figure A2:**
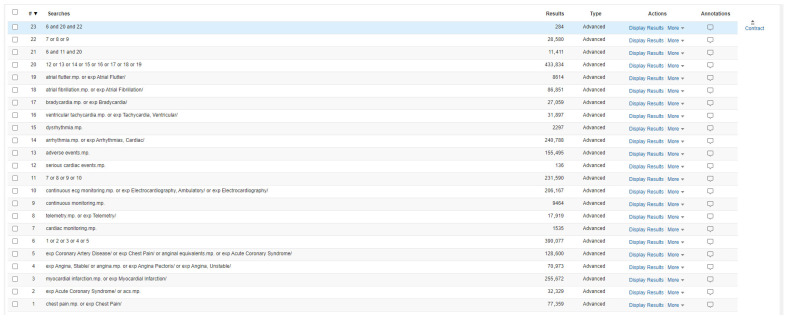
Search strategy utilized for the OVID Medline 1946—present with preprint database.

Evidence surrounding the use of CCM in the emergency-chest-pain population was obtained through a comprehensive literature review (search strategy can be found in Appendix A: Figure A2). From this, a two-step decision tool evaluating patient ECG and chest pain was created through an iterative process with a third step; initial cardiac biomarker, being added for future use in a separate initiative. The tool delineates if a patient needs transport by EMS or if safe for non-monitored transport. This was specific to patients being evaluated for ACS rule-out and deemed low risk by the referring physician. Exclusions included the following:

- transfer for alternative work-up and diagnosis (i.e., not for second serial troponin)
- hemodynamic instability
- arrythmia at any time during UCC stay
- physician gestalt

#### 2.3.2. Developmental PDSA—Feasibility Testing—Chest-Pain Monitoring Transport Tool

**Figure 1:**
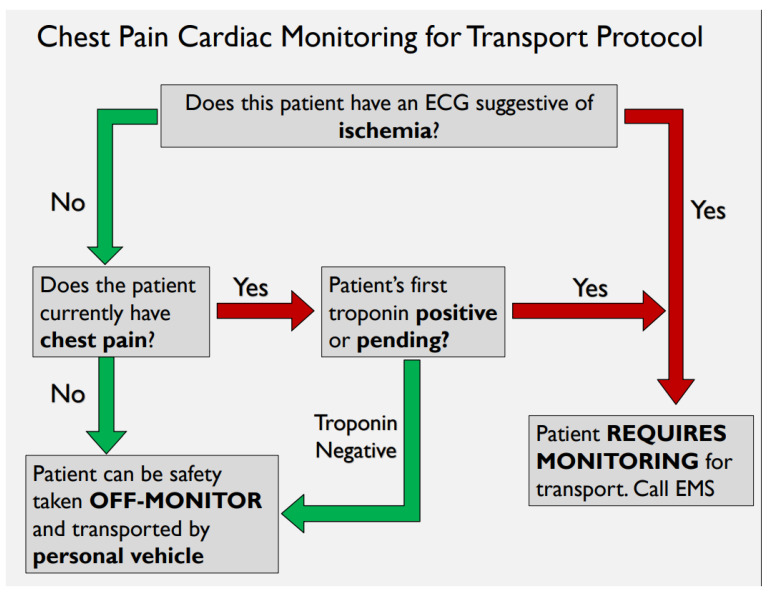
Safe-for-self-transport CDT. This was adopted by the clinical group. Red arrows would indicate the need for monitoring on transport, whereas the green arrow pathway indicates safe for off-monitor.

Following development of the “safe-for-self-transport” CDT, two variations were provided to 11 resident physicians. A series of 5 clinical test scenarios were completed using each tool. In addition to whether residents answered the clinical scenarios correctly, participants were asked a short series of questions surrounding the aesthetics of each tool, the ease of function and their overall preference if using clinically. The results of this feasibility study led to the adoption of Figure 1 (below) as the “safe-for-self-transport” chest-pain transport tool implemented in this study.

#### 2.3.3. Implementation PDSA—Educational Session and Chest-Pain Monitoring Transport Tool

Implementation occurred in the usual fashion with departmental notification of its impending usage and educational rollout. This was followed by a soft-launch period where usage was encouraged but not mandated to familiarize oneself with the CDT. Lastly, formal CDT adoption as standard work by our physicians occurred with mandated usage and EMR integration.

### 2.4. Measures

#### 2.4.1. Main Outcome Measures

1. Proportion (%) of patients presenting with chest pain to UCC transported by EMS to the main hospital for repeat troponin (bi-weekly).

#### 2.4.2. Fidelity/Process Measures

1. Proportion (%) of eligible patients where the transport monitoring tool was documented as applied (bi-weekly).

#### 2.4.3. Balancing Measures

1. Time to second troponin draw in minutes (comparison between self-transported patients before and after intervention bundles).
  - Concern that patients self-transporting would not receive their second troponin biomarker blood draw within the normal timeframe as this proportion of patients theoretically increased.
2. Proportion (%) of patients self-transported that were admitted.
  - Increasing admission proportion among self-transported patients could indicate misclassification of low-risk patients and unsafe use of tool.
3. Proportion (%) of self-transported patients that had an increased Canadian triage acuity score (CTAS) on arrival to main hospital
  - By looking at the change in score between UCC and the main hospital, we can infer if self-transported patients were presenting as sicker to the main hospital. In theory, this would also capture if these patients suffered an adverse event on transport necessitating a higher acuity triage on arrival to the main hospital

### 2.5. Analysis

Primary outcome measures were analyzed with the appropriate statistical process control charts. Control limits were fixed around the baseline data acquisition period and post-intervention data was analyzed for established Nelson, Western Electric and Shewart special cause variation rules; shifts, trends, astronomical data points and the two-out-of-three rules [12,13,14,15,16]. The statistical approach used in this study is best explained and summarized by Benneyan et al. 2003, but further detail is available within The Healthcare data guide: learning from data for improvement by Prevost [13,15]. P-charts were chosen as the main SPC chart for the primary outcome through the statistical software; (Version 2021.09). Non-paired two-tailed *t*-tests were performed to compare differences between self-transported and EMS-transported groups of patients for all balancing measures listed.

## 3. Results

### 3.1. Outcome Measure

**Figure 2:**
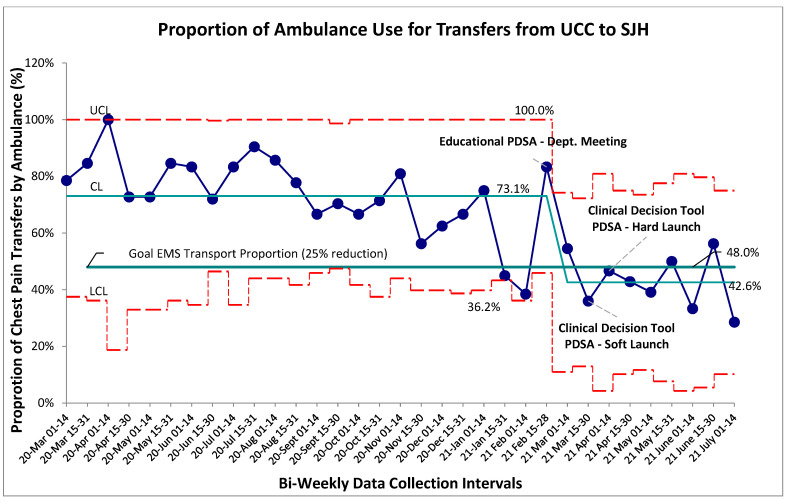
Proportion of EMS use for UCC to SJH Transfers. SPC–P chart showing continuous time interval data of the proportion of patients with a chief complaint of chest pain transported with EMSs. Study interventions include educational review of the cardiac monitoring literature, followed sequentially by soft- and hard launches of the clinical decision tool. Special cause variation identified beginning with the 15–28 February data point. The red lines represent the upper control limits (3SD), while green line represents goal target mean.

Baseline data for the proportion of patients with chest pain transferred from UCC to the main hospital by EMS was collected and aggregated bi-weekly through an 8-month retrospective review followed by a 4-month prospective review. Stable common cause variation was found within this system with a mean proportion of EMS transfers for patients with chest pain established at 73% (Figure 2). No established special cause variation was identified within the baseline system. A package of interventions was introduced in rapid succession beginning with an educational PDSA, departmental adoption then staged rollout of the “safe-for-self-transport” CDT. Following initial educational PDSA all prospective post-intervention data points were found to be below the previous control limit (mean) indicating a shift and establishing special cause variation secondary to the implemented PDSAs. New baseline control limits were established for this post-intervention system at 43% (Figure 2). Given the sequential roll-out of three system changes over a short interval within this special cause variation, it is difficult to identify one single contributing factor to the overall successful reduction in proportion of EMS transfers from UCC to the main hospital. Educational interventions are typically prone to the Hawthorne effect and eventual regression to historical baselines, making it more likely that a department-adopted CDT played a significant role in the 14-week post-initial-intervention bundle success. We recognize that the contributions of educational sessions and the CDT cannot be easily defined by the structure of this study.

### 3.2. Fidelity (Process Measure)

**Figure 3:**
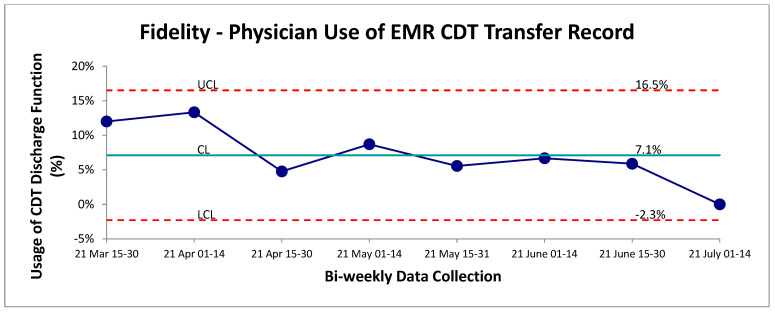
Physician adherence to tracking procedure for use of CDT. SPC—chart P continuous data evaluation of physician usage of discharge button indicating which type of transport (self vs. EMS) was selected on the EMR. Failure to use this EMR feature effectively does not necessarily mean they did not apply the CDT; this feature was developed as a proxy for tracking. Red lines are the upper and lower control limits (3SD) and green line represents mean control limit.

Adherence to the CDT was monitored through the addition of a new EMR discharge order button designed for physicians to track their usage. The lack of using this specific order did not explicitly mean the tool was not used. Overall, this function appeared to be minimally utilized by staff physicians following the transport of a chest-pain patient from UCC to the main hospital. The mean adherence was established at 7% over the post-intervention period (Figure 3). Given the positive outcome measure results, this would be discrepant with this fidelity measure. One likely scenario is that physicians were either unaware of this discharge tracking metric or found the extra search and click for this specific discharge button too cumbersome to be utilized effectively in a busy clinical setting. Further root-cause analysis will be required to optimize this process and ensure accurate tracking of CDT usage.

### 3.3. Balancing Measures

**Figure 4:**
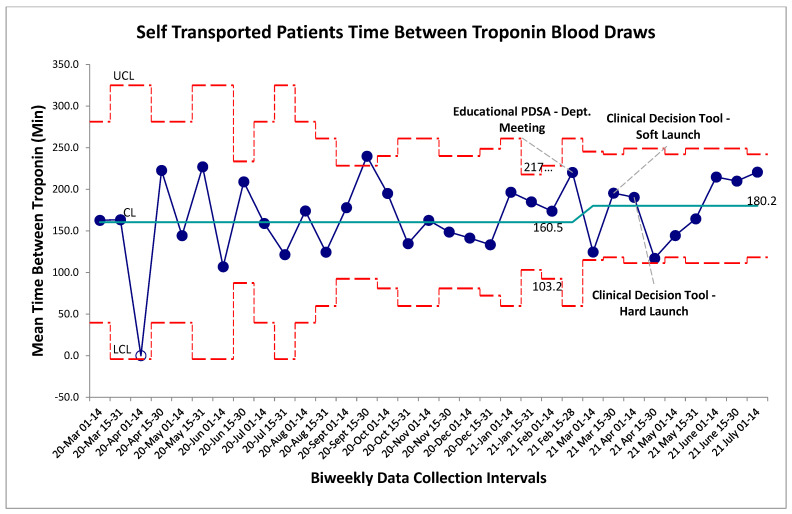
Self-transport patient time between cardiac troponin biomarker bloodwork. SPC X-bar chart identifying continuous time interval data for the time between evaluation of cardiac biomarkers within the self-transported chest-pain patients. No special cause variation identified. Red lines represent upper and lower control limits (3SD) while green represents mean control limit.

**Figure 5:**
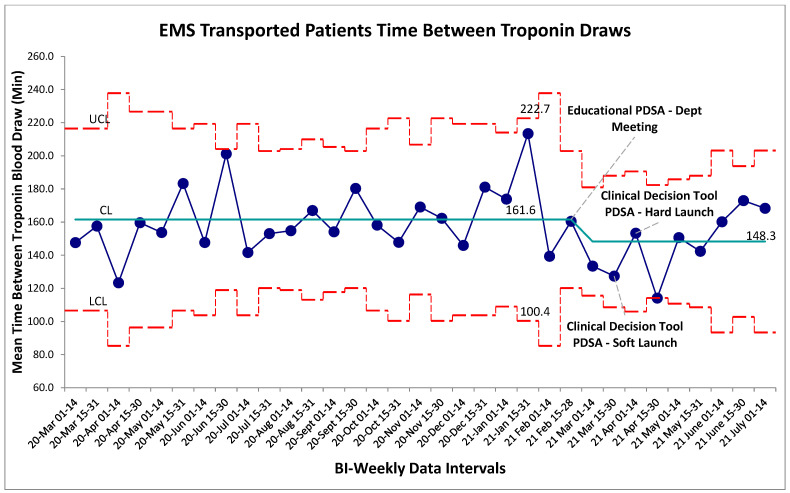
EMS transported patient time between cardiac troponin biomarker bloodwork. SPC X-bar chart identifying continuous time interval data for the time between evaluation of cardiac biomarkers within the self-transported chest-pain patients. No special cause variation identified.

Balancing measures for unintended harms were tracked through three separate metrics for this study, the first being time between troponin blood draws for self-transported patients. The established gold-standard is a repeat troponin biomarker test 180 min following the initial blood draw. Within the baseline data collection period, mean time-between-troponin (TBT) was established at 160 min. Following the series of interventions the post-intervention mean was found to be 180 min. Within the post-intervention period, new control limits were calculated for visual representation on the graph (Figure 4); however, no special cause variation was identified to suggest a deviation from a stable system within this timeframe. Non-significance was additionally established in pre/post-comparison between aggregate baseline and post-intervention data (*p* > 0.05, two-tailed *t*-test), further establishing that the intervention package did not alter TBT. For comparison, the TBT for EMS transferred patients was also calculated which also revealed no special cause variation post-intervention (Figure 5). Given that the intervention should not have altered the pathway by which EMS-transferred patients receive bloodwork, this result was expected.

**Figure 6:**
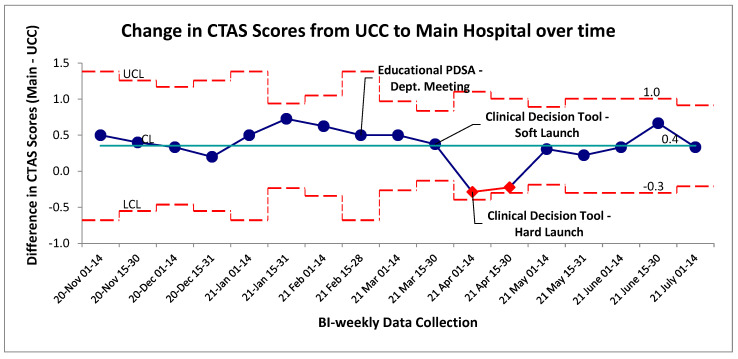
Change in CTAS scores between UCC arrival and main hospital arrival in self-transported patients. SPC XBar chart illustrating trends over time to CTAS scores. Red data points represent special cause variation in the system, red lines represent upper and lower control limits (3SD), green line represents mean control limit.

Changes in CTAS scores were evaluated as a surrogate for changes to self-transported patient acuity level. The baseline changes in CTAS scores from UCC to the main hospital with a negative score indicated a more acute presentation upon arrival to the main hospital, and a positive change meant the patient was scored as a less acute presentation (Figure 6). Special cause variation was identified post-intervention for an established period between 1 and 31 April 2021 by the two-of-three rule. When evaluating these two data points at a patient level, this special cause variation was driven by a portion of the patients within these four weeks being scored as CTAS 4 and 5 (the lowest acuity) at UCC and scored as a 2 or 3 at the main hospital site. Had they been scored appropriately for an initial chest-pain assessment, their score would have remained unchanged between the two sites and this variation would not have occurred. The overall mean change in CTAS score was similar pre/post-intervention (Figure 6). Tradition pre/post aggregate statistical analysis showed no significant difference between the two groups (*p* > 0.05, two-tailed *t*-test).

**Figure 7:**
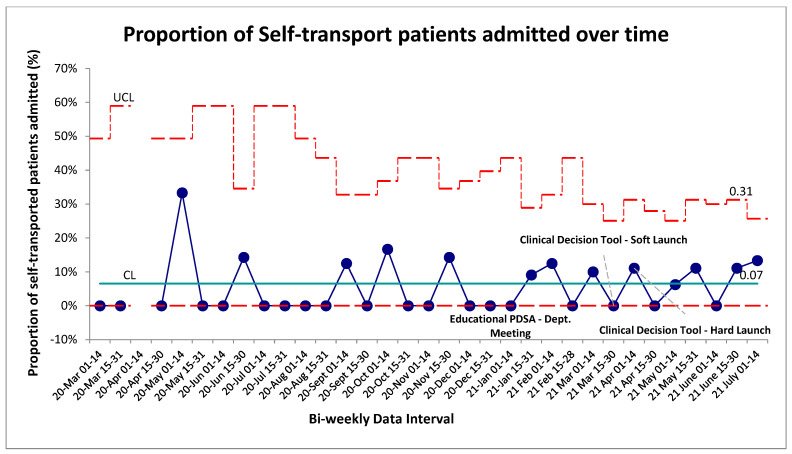
Admission rates of self-transported patients over time. SPC—X chart illustrating trends to the % of patients admitted following troponin evaluation and transfer over the bi-weekly data collection period. Red lines represent upper and lower control limits (3SD) while green line represents mean control limit.

As the CDT and intervention package were intended to identify low-risk patients for transport, the admission rate of self-transported patients was tracked over the baseline and intervention timelines. This was used as a surrogate to ensure that as the proportion of self-transported patients increased, the overall illness characteristics of the self-transported population did not change and that the tool was not inappropriately circumventing clinician gestalt of identifying sicker patients. The baseline admission rate of this self-transported chest-pain population was established at 7% (Figure 7). In the post-intervention period, no special cause variation was identified, indicating no change in the overall system from the implementation of the CDT. In an aggregate pre/post analysis the post-intervention admission percentage of self-transported patients was 6%. No significant difference was found between the two groups (*p* > 0.05, two-tailed *t*-test).

## 4. Discussion

Chest pain is a common presentation to the emergency department, and the majority will require and undergo a cardiac work-up, ultimately to be discharged home with a negative result. The widespread uptake of CDTs such as the GRACE, TIMI and Heart Score aimed at early discharge within this population has been limited, owing to the varying degree of re-visit rates with the potential for major adverse cardiac events above the 1% threshold that most clinicians feel is an acceptable risk rate [2,17,18,19]. While recent scores such as the Heart Pathway and Clinical Chemistry Score (CCS) show more promising risk profiles aimed at early discharge, they have not made their way into guideline-based practice as of yet [3,4,20,21,22,23]. This risk stratification dilemma is magnified in centers such as our urgent care facility, a 65,000 patient-visit facility that has no admission capacity and limited laboratory assessment for guideline recommended serial high-sensitivity troponin biomarkers: necessitating the completion of the cardiac work-up for these patients to be performed across the city at the main hospital site. Furthermore, the main mode of timely transportation relies on EMS, a finite resource that often faces significant patient off-load times—rendering them inoperable until the patient can be successfully delivered to a care area under the hospital responsibility. Given the low overall prevalence of disease in the transported population of chest-pain patients, this interface represented a key area for adaptation of guidelines and literature to reduce transport burden for EMS, while still safely and effectively identify patients able to transfer by personal means.

The AHA has a COR 1 (level b) recommendation that anyone who is intermediate- or high-risk for ACS requires ongoing CCM. The recommendation drops to CORIII (level c) evidence in patients with normal ECG and negative biomarker evaluation for arrhythmia and CORIII for low-risk and noncardiac chest pain or patients that are fully awake and able to verbalize changes in symptoms [1,7]. This guideline resonated with a key factor of the decision for EMS usage of patient transport found within the data drill down—the perceived need of CCM. However, these guidelines are based largely on inpatient populations and older data and did not necessarily reflect the latest emergency-medicine literature.

Focusing on the low-risk chest-pain population, an absence of new ischemic changes on ECG and resolution of chest pain was validated as a safe and effective means to remove CCM in patients whose initial complaint to the ED was chest pain [5,6,10]. Given that these patients had no monitoring for their entire ED stay and work-up (multiple hours), we felt that this would be a safe and effective tool to adapt as a “safe-for-self-transport” CDT. Following a usability study with clinical scenarios for two versions of the tool that differed in appearance, we were able to implement a quality-improvement bundle including education, and this CDT successfully reduced the proportion of patients transferred by EMS by 30%, achieving the initial goal of a 25% reduction in transfers.

The balancing measures were chosen largely to ensure patient safety was not compromised through the initiation of this CDT. Firstly, the research group needed to be sure that patients were still meeting appropriate gold-standard benchmarks for time-between-troponin within their chest-pain evaluation. The goal was that with the increased proportion of self-transported patients, their second troponin biomarker needed to be drawn within historical limits. No change in the system on SPC charting or in pre-post analysis was detected in this measure, indicating that the tool did not alter this standard of care. Secondary to this was the need to identify any patient that was self-transported inappropriately. The combination of the change in individual CTAS scores of self-transported patients again revealed a safe system within the study period with no compromise to patient safety or misidentified patients. In the 186 self-transport patients discharged from ED on index visit, only 6 had repeat visit within 72 h and none for sentinel diagnoses requiring admission.

A post-intervention admission rate of 6% within the self-transported population indicated a stable system despite the significant proportional increase in self-transported patients. This surrogate marker helped to alleviate staff physician concerns that the tool would be imperfect for identifying low-risk patients safe for transfer. Prior to the initiation of the new transport process, transport via EMS was performed for several reasons, but ultimately with no formal process, it meant clinician gestalt was the only clinical factor for this type of transport. With the post-intervention baseline possessing no special cause variation and the post-intervention admission rate remaining stable, it helps to solidify that changing the transfer process did not impact clinical gestalt for identifying patients that require higher levels of care and inpatient work-up.

### 4.1. Future Direction

The main direction of this project focuses on another aspect of the tool’s intended use. The results of this quality-improvement initiative were based on physician education and the two-step process found on the CDT. Following the literature and guideline review, the patient’s initial cardiac biomarker status (negative or positive) was added to the CDT, as evidence suggests a negative initial troponin biomarker also renders a patient safe for off-monitor evaluation [8,9]. The UCC clinicians will not have this information prior to planning for transport, so this portion of the CDT did not apply to this study but is intended toward adaptation for the triage nurse at the receiving main hospital to fast-track the offload of any EMS-transported patients should their troponin be negative. We hope to implement a process in which these patients can be offloaded from EMS responsibilities to the waiting room, thus improving these offload times based on the patient’s initial ECG, presence of chest pain or verification of a negative initial cardiac biomarker. The other avenue which has been explored by the research team but is not currently feasible due to financial constraints is the addition of a point-of-care troponin machine

### 4.2. Limitations

There are a few limitations to the evaluation within this study. The first is that the change in the CTAS score and change in the admission rates are both surrogates for understanding whether the self-transported patient was inappropriately selected for self-transport over EMS. However, because these are surrogate markers, they remain imperfect for identifying whether a self-transported patient could have significantly deteriorated on transfer. There were no reported events throughout the duration of this study or found within chart review. It is well established in Canadian emergency-medicine literature that patients who are chest-pain free with a normal or non-specific ECG changes suffer no major adverse events such as arrythmia while awaiting work-up without ongoing telemetry monitoring [5,6]. The low-risk population within this study mirrors this pre-established population that does not require ongoing monitoring for their stay. Furthermore, we recognized that the interrater reliability of CTAS score is imperfect, and thus further safety monitoring should be explored on an individual patient-level [24].

With respect to the ongoing evaluation of the new transfer process, there does appear to be a significant issue with confirming use by the physicians as evidenced by the fidelity measures (Figure 3). The mechanism of monitoring was achieved solely through an additional discharge button integrated in the EMR. Unfortunately, this did not undergo human-factor design or testing by the physician group prior to its implementation, and it appears that it was not effectively utilized. The research group feels the actual fidelity and usage of the new transfer process is higher than this measure indicates for two reasons. First, the average number of patients transported bi-weekly increased in the post-intervention period, and the average number of self-transported patients doubled in the post-intervention period. While this could solely be due to the physician-education component of the new process, it seems unlikely as it was a non-mandatory education session attended by approximately 50% of the total physician pool. Secondly, for all patients that were being transported, nursing was aware of the intervention and had to confirm the method of transportation, acting as a secondary reminder to the physicians. Nevertheless, future direction would include addressing the lack of usage of this button and standardizing the reported transportation method on triage to the receiving hospital to ensure viable tracking.

Logistical challenges for patient self-transport remain an issue. When a patient is transported by EMS, the hospital is not responsible for the cost of the transport, the healthcare system and patient are. In the event of the hospital asking the patient to take a taxi or have a relative transport them, the hospital is either placing the cost back on the patient or absorbing that cost itself, which may create a barrier to care for some patients. It is important to point out that the cost of a “medically necessary” EMS transfer in Ontario, Canada is $45.00 billable to the patient and “medically unnecessary” $245.00. Many of these patients being discharged from the ED would mean they fall into the latter category. The average taxi fare for the same distance is approximately $25.00 and therefore more economical. If a patient was deemed suitable for self-transport and did not have a family member with them, paid taxi vouchers were available at physician/nursing discretion. If a physician felt that it was in the best interest of the patient to transport by healthcare professionals (i.e., mobility issues), traditional EMS transport was utilized. We recognize that there is complexity in this decision making beyond strictly medical considerations.

Lastly, as an uncontrolled time interrupted quality improvement study, it can be susceptible to temporal biases. There were no obvious changes to the structure of the departmental or regional policies, personnel, or EMS protocol that would have significantly contributed to bias. However, we do recognize that an underappreciated external factor could have contributed without our knowledge.

## 5. Conclusions

EMS remains a finite resource within many Canadian health regions. By focusing on a cardinal emergency-department presentation of chest pain, our team was able to identify the perceived need for cardiac monitoring as a main root-cause contributor to the excessive use of EMS for interfacility transport and create targeted solutions. Through dedicated adaptation of a comprehensive literature review, we were able to reduce the proportion of chest-pain patients being transferred using EMS by 30%, achieving our modest goal of 25% over the study period. A quality-improvement initiative centered around the implementation of a safe-for-self-transport CDT has led to sustained system change with no increased evaluation time, acuity, or admission rate within the self-transported population. Given this tool’s components have been previously validated in other health regions, the generalizability of this intervention to other interfacility, inter-city transport settings remains likely. Further optimization and ongoing evaluation of this CDT and the transport process of these patients has the potential to safely reduce both EMS transport and patient offload further, reducing overall offline time for EMS and improving EMS access.

## Data Availability

Data can be made available to those interested through email from the corresponding author.
